# Scanning behaviour in ants: an interplay between random-rate processes and oscillators

**DOI:** 10.1007/s00359-023-01628-8

**Published:** 2023-04-24

**Authors:** Sudhakar Deeti, Ken Cheng, Paul Graham, Antoine Wystrach

**Affiliations:** 1 School of Natural Sciences, Macquarie University, Sydney, NSW 2019 Australia; 2 School of Life Sciences, University of Sussex, Brighton, UK; 3 Centre de Recherches Sur La Cognition Animale, CBI, CNRS, Université Paul Sabatier, Toulouse, France

**Keywords:** *Melophorus bagoti*, View-based navigation, Poisson process, Saccadic scanning

## Abstract

At the start of a journey home or to a foraging site, ants often stop, interrupting their forward movement, turn on the spot a number of times, and fixate in different directions. These scanning bouts are thought to provide visual information for choosing a path to travel. The temporal organization of such scanning bouts has implications about the neural organisation of navigational behaviour. We examined (1) the temporal distribution of the start of such scanning bouts and (2) the dynamics of saccadic body turns and fixations that compose a scanning bout in Australian desert ants, *Melophorus bagoti*, as they came out of a walled channel onto open field at the start of their homeward journey. Ants were caught when they neared their nest and displaced to different locations to start their journey home again. The observed parameters were mostly similar across familiar and unfamiliar locations. The turning angles of saccadic body turning to the right or left showed some stereotypy, with a peak just under 45°. The direction of such saccades appears to be determined by a slow oscillatory process as described in other insect species. In timing, however, both the distribution of inter-scanning-bout intervals and individual fixation durations showed exponential characteristics, the signature for a random-rate or Poisson process. Neurobiologically, therefore, there must be some process that switches behaviour (starting a scanning bout or ending a fixation) with equal probability at every moment in time. We discuss how chance events in the ant brain that occasionally reach a threshold for triggering such behaviours can generate the results.

**Supplementary Information:**

The online version contains supplementary material available at 10.1007/s00359-023-01628-8.

## Introduction

Inspired by the work of Wehner and many collaborators, desert ants have become well known for their navigational prowess (Wehner 2013, 2020). The ants’ navigational toolkit contains path integration (Collett and Collett 2000; Wehner and Srinivasan 2003), view-based navigation (Wehner 2003; Knaden and Graham 2016; Freas and Cheng 2022), systematic search (Wehner and Srinivasan 1981; Schultheiss et al. 2015), and backtracking (Wystrach et al. 2013; Freas et al. 2019). In path integration, the ant keeps track of the distance and direction from its starting point. Some of the behavioural and neural mechanisms of path integration have been worked out (Stone et al. 2017; Heinze et al. 2018). In view-based navigation, ants use the panoramic view to navigate (Wystrach et al. 2011a, 2011b), and some of the features of views that they use are known, such as the skyline (Graham and Cheng 2009) and the fractional position of mass (how much of the scene is to the right vs. left of the target direction of travel; Lent et al. 2013). Neurobiologically, the use of views is dependent on the mushroom bodies. Silencing of the mushroom bodies degrades view-based navigation in two different species of ants (Buehlmann et al. 2020; Kamhi et al. 2020). In systematic search, ants loop around the a focal point in loops of increasing size (Schultheiss et al. 2015; Waldner and Merkle 2018). And backtracking takes place if an ant that has reached a location near its nest is displaced to an unfamiliar location. The ant heads off, at least for a short distance, in the direction opposite to the route-based feeder-to-nest direction, as if assuming that it has overshot its familiar route corridor (Wystrach et al. 2013; Freas and Spetch 2021). Both systematic searching and backtracking depend on path integration to function. Path integration is thought to be always at work in the background even if other strategies are being pursued (Wehner 2020).

In using views to navigate, ants must learn views around their nest. They do this by a series of exploratory walks executed before they head off to forage, so-called learning walks (Fleischmann et al. 2016, 2017, 2018a, b; Jayatilaka et al. 2018; Deeti and Cheng 2021a; reviews: Freas et al. 2019; Zeil and Fleischmann 2019; Fleischmann et al. 2020). Similar to systematic search, loops of increasing size are performed over successive trips. During these trips, ants make frequent rotations with body saccades to face different directions briefly, the entire bouts being called pirouettes (Fleischmann et al. 2017), although the extent to which the head is immobile in these saccades has been questioned (Zeil and Fleischmann 2019). In *Cataglyphis nodus* exiting the nest for the first times, the directions in which they face are determined by a path integration mechanism based on geomagnetic cues (Fleischmann et al. 2018a). Pirouettes are thought to help the future foragers to learn views facing the nest from different directions as well as other views.

Desert ants also pirouette and take fixations occasionally during normal foraging journeys (Wystrach et al. 2014). This is especially prominent at the beginning of journeys home, when ants have to find the best travel direction. Scans increase in number after failures in navigating a familiar route (Wystrach et al. 2019, 2020a) or when the scene has been experimentally altered (Wystrach et al. 2014; in bull ants: Narendra and Ramirez-Esquivel 2017; Islam et al. 2020, 2021). This important behaviour in the toolkit of ants has not been well characterized, and we begin to quantify scanning bouts in this account, concentrating on the timing of behaviours with a view to what we can learn about the neural organization of scanning.

We made homing red honey ants, *Melophorus bagoti*, come out of a short channel—which blocked the surrounding terrestrial visual information—before they set foot on the natural sandy terrain with a full panoramic view. This made the ants scan in pirouettes before marching off. We videotaped this initial stage at 300 frames/s to detect fixations, when the ant was truly immobile, to examine (1) how long fixations last, and (2) how long intervals between scanning bouts are. These inter-event intervals inform us about the organization of behaviour, with implications for underlying neural processes. We formulated three hypotheses to test before launching into the data analysis.

One hypothesis is that the inter-event intervals (fixation durations and inter-scanning-bout intervals) are periodic, giving rise to a Gaussian distribution of inter-event intervals peaking at the period of the cycles, with the Gaussian spread representing the imprecision in the system. This pattern implies an oscillatory system that generates neural pulses and downstream behaviour on a regular periodic basis (Gallistel 1980). These oscillators act at different scales. For instance, insects such as ants (Clement et al. 2023), moths (Namiki and Kanzaki 2016) or *Drosophila* larvae (Wystrach et al. 2016) possess such intrinsic neural oscillators in their brain’s pre-motor area (Steinbeck et al. 2020) to produce slow (around 1 Hz) but regular lateral zigzags along their paths. These oscillations participate directly in the navigational task (Baker and Vickers 1997; Lent et al. 2010; Kodzhabachev and Mangan 2015; Murray et al. 2020; LeMoël and Wystrach 2020; Wystrach 2021). At the scale of step-by-step locomotion as well, walking insects typically orchestrate their six legs as coupled oscillators—likely following commands from the thoracic ganglia (Steinbeck et al. 2020) and acting at high frequency (> 10 Hz) to carry out the common tripod gait (Wilson 1966; Gallistel 1980; Pfeffer et al. 2019; Tross et al. 2021). In this gait, the front and rear legs of one side are teamed with the middle leg of the opposite side (forming a tripod when they land approximately together). Oscillators in neurally endowed animals are based on pacemakers (Gallistel 1980; Marder and Bucher 2001), circuits or single neurons that pulse regularly to cause periodic downstream behaviour. Could a pacemaker-based oscillatory system drive fixation durations and inter-scanning-bout intervals?

A second hypothesis is in a sense the opposite of an oscillatory process: a random-rate or Poisson process, which is as irregular as can be. In a random-rate process, the probability of a type of event taking place (e.g., stopping a fixation and initiating a body saccade) is constant at every moment in time. Inter-event intervals are therefore ‘blippy’ and ‘gappy’, with a concentration of short intervals. The signature for a random-rate process is an exponential distribution of inter-event intervals. Some comparative evidence points to such random-rate process in the interruptions to forward movement, both in non-neural organisms (bacterium *Escherichia coli*, Berg and Brown 1972; Cheng 2022) and in small-brained animals (nematode *Caenorhabditis elegans*, Srivastava et al. 2009; Cheng 2022). Could a random-rate process be involved in generating scans in desert ant navigation?

A third hypothesis that we entertained is that the temporal distributions might be akin to a Lévy walk as seen in search behaviour. A Lévy walk is called heavy-tailed because it has a higher proportion of very long straight lines (Reynolds 2018), which, in our current study, translates to long temporal durations or inter-event intervals. The classic form is a power-law distribution of inter-event times. In systematic search, however, ants do not do Lévy walks (Reynolds et al. 2013; Schultheiss et al. 2015) but manage to approximate a Lévy walk by adding multiple exponential distributions together, indicative of random walks at different scales. We tested a distribution that resembles what would be produced by a Lévy-like process called the stretched exponential (Ferdous et al. 2018; details in the Materials and Methods). Would a Lévy-like process characterize the temporal distributions seen in scanning?

## Materials and methods

### Animals and experimental site

The experiments with Australian desert ants *Melophorus bagoti* were conducted during the summer months from January to March 2010 on a field site in the outskirts of Alice Springs, Northern Territory, Australia with various tussocks of buffel grass (*Pennisetum cenchroides*) interspersed with *Acacia* spp (Deeti and Cheng 2021b) and occasional large eucalyptus trees and bushes. *M. bagoti* are widespread over the area, forming underground monodomous colonies with typically only one entrance on the ground. The thermophilic red honey ant *M. bagoti* forages during hot periods of summer days, mainly scavenging dead arthropods and gathering sugary plant exudates and seeds (Muser et al. 2005; Schultheiss and Nooten 2013). To conduct research on ants, Australian ethical approval is not required.

### Feeder and channel set up

**Fig. 1:**
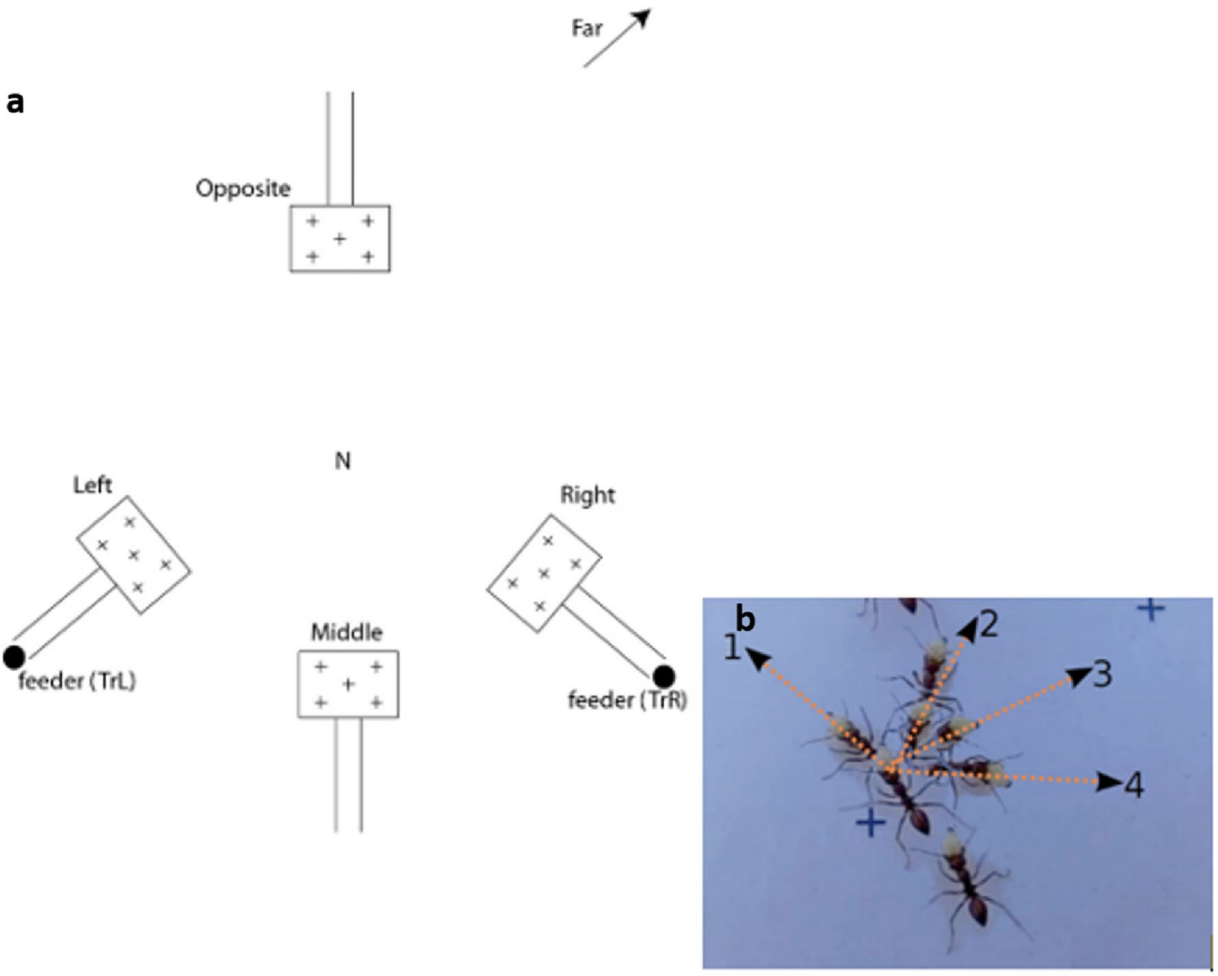
Schematic representation of experimental set up and dependent measures. **a** The feeders were 5 m from the nest (N). The sets of two parallel lines represent a channel (open to the sky) that the ants had to travel through. The rectangles with + s represent a white sheet of paper for videotaping purposes. The + s represent marks on the sheet of paper. The Far location, approximately 40 m away from the nest, had a similar channel-and-sheet set up oriented in the same direction as the Opposite location. Not to scale. **b** An example of a scanning bout taken from video frames. In this bout, the ant stopped first facing direction 1, then turned to directions 2, 3, and 4 in turn, each time remaining immobile for a short duration

**Fig. 2:**
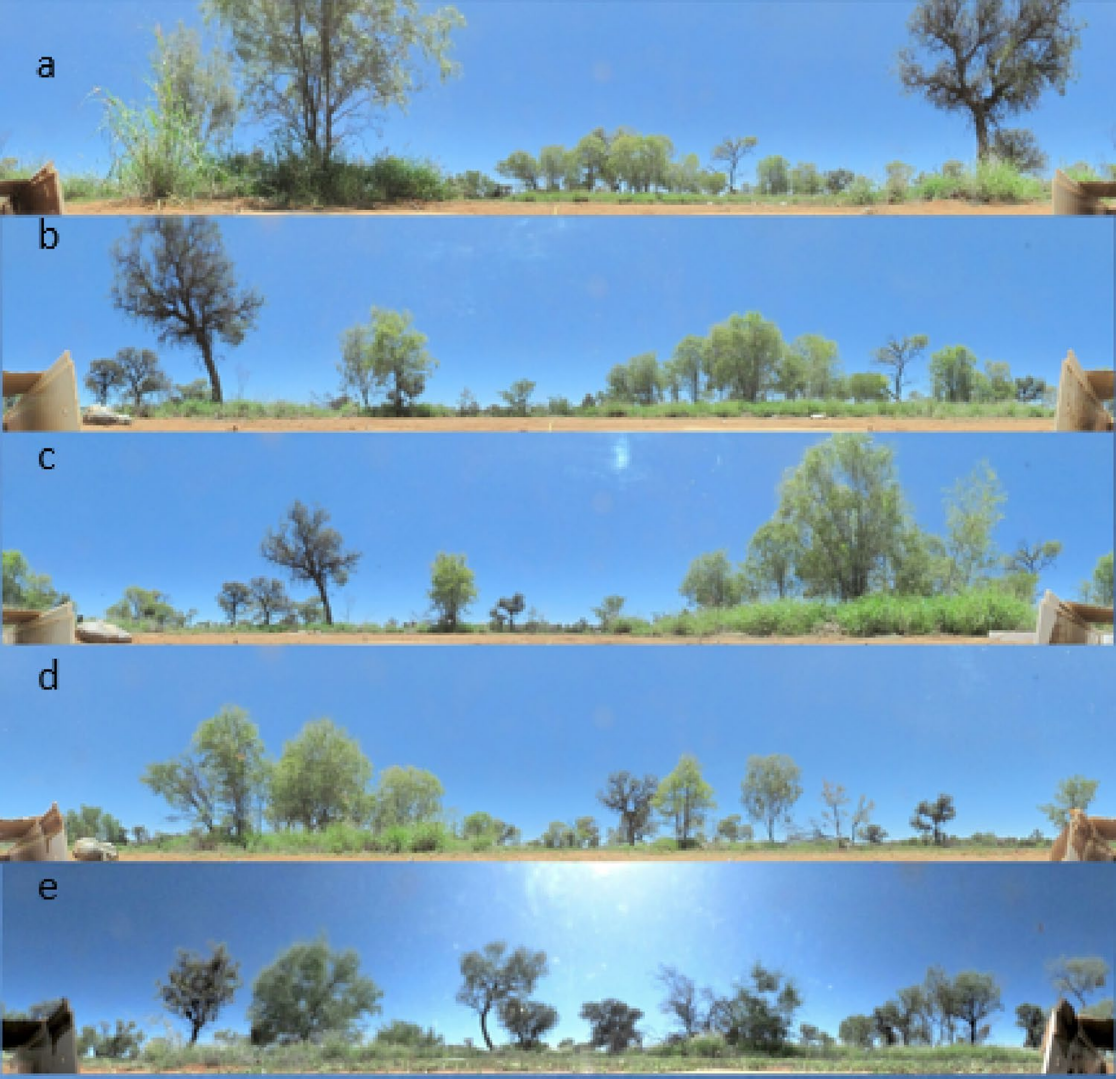
Panoramic 360° images of the five different test sites. Images were taken with a Sony HD bloggie camera and unwrapped into 360° cylindrical images. The Left **a**, Middle **b**, Right **c**, Opposite **d** and Far **e** test-site images were taken from the centre of sheet of paper placed in the video-recording arena just outside the channel exit

The experiment was conducted on a single nest of *M. bagoti* (colony location at 23°45.448′ S, 133° 52.908′ E). The nest area was cleared of grass but surrounded by bushes and trees. We buried two feeders (15 × 15 × 15 cm box with slippery surface) at ground level, 5 m from the nest in directions 120° apart (Figs. 1a and 2), called Right and Left locations. The feeders could be filled with cookie pieces (see Procedures) to attract foraging ants. Each feeder was connected to a 1 m long, 10 cm wide, and 10 cm high channel pointing towards the nest direction (Fig. 1a). The channels were buried 10 cm into the ground at the feeder end, providing a smooth slope up to the surface on their exit end. A small wood ramp enabled the ant to exit the feeder and jump directly into the channel. At the other end of the channel, a wooden board 60 cm wide and 120 cm long was laid on the ground, levelled with and connecting to the channel floor. The ant would thus exit the channel onto this board and perceive the surrounding natural scene. An A4 sheet of white paper was glued on the board, providing contrast for video recording the ants’ movement right after they exited the channel. Five discrete symbols were printed on the sheet of paper to enable calibration when subsequently video-tracking the ant movements. We set up similar channel constructions at the three other locations called the Middle, Opposite, and Far locations (Figs. 1a and 2) for testing. These locations had no feeder. During tests, ants were released directly into the channels.

### Procedures

We first provided cookies (Arnott brand) at the Right feeder only (Fig. 1a). All foraging ants arriving at the feeder were marked with a dot of paint of one colour (Tamiya^™^) on their gaster to indicate that they had been at least once to this feeder. For training, these marked ants were allowed to visit the feeder and exit through the channel with a food crumb multiple times for 2 full days. To ensure that only experienced ants were tested, we selected only painted foragers that came to the feeder and went back to the nest in straight, unhesitant trajectories. For tests, an experienced ant homing with its cookie crumb was gently captured just before entering its nest, thus depriving it of path integration information (Cheng et al. 2009). It was then placed at the feeder end of one of the channels. The captured ant would typically run up and exit the channel, where it would be video recorded, and then be caught again after exiting the wooden board. That way, each ant could be tested four times in a row at the Left, Middle, Right, and Opposite locations (Fig. 1a). The order of release locations was counter balanced across individuals. Only ants that held the food throughout the entire procedure were included for data analysis, as this indicates that the individual was continuously motivated to carry the morsel back to its nest.

Once 48 ants had been successfully tested (which took 3 days), we shifted the training location to the Left feeder: The Right feeder was emptied, and the Left feeder was filled with cookie crumbs. All previously painted ants with familiarity with the Right feeder were henceforth ignored. We painted a new cohort of ants using a different colour—these individuals were thus naïve of the Right feeder but had discovered the Left one—and let them train for two full days and repeated the test procedure until 48 ants had been successfully tested (which took 4 days).

Finally, as an additional test condition, we set-up a fifth channel at a distant location called the Far location. This channel was oriented in the same compass direction as the Opposite channel, but located around 40 m away behind a barrier of bushes and trees, and thus providing an unfamiliar scenery to the ants. We again trained new cohorts of ants to the Left feeder (*N* = 14), and then to the Right feeder (*N* = 19), and tested experienced individuals at the Far location only, following the same test procedure.

For all tests, we used a high-speed video camera (Casio Exilim EX-F1, Casio Computer Co., Ltd.) to record the ants exiting the channel over a 30 × 30 cm area at 300 frames/s. The camera was mounted on a tripod which we shifted across test locations to stand in the same position relative to the exit set up, providing a slight modification of the natural panorama, a change that was similar for all tested ants at all test locations. During training, the tripod and camera were at the training-channel location.

### Data analysis

We carried out frame-by-frame analysis to determine head and body orientation. We isolated the moments where the ant stayed in the same location on two consecutive frames. This indicates a pause in both forward motion and body rotation, which we call a “fixation” (Fig. 1b). For all fixations, we extracted the ant’s body orientation and position, based on the head and pronotum, using a custom-written MATLAB-based algorithm (Matlab R2010a, Mathworks). The middle of the channel exit location was chosen as the origin (0, 0). As reported before (Wystrach et al. 2014), this species tends to display a series of successive fixations in different head directions by rotating on the spot at one location, which we call here a “scanning bout”. In other words, a scanning bout is a collection of fixations displayed at one spot before an ant resumes forward motion. For each trial, we extracted the number of scanning bouts, the inter-scanning-bout intervals (time since the start of the previous bout, not available for the first bout), the scanning-bout durations (from the start of a scanning bout until the ant starts walking again), the number of fixations per bout, and all turn angles and fixation durations. The turn angle, measured within each bout, is the directional difference between the orientation of fixation i + 1 and fixation i, for i = 1 to *n*−1 (n being the total number of fixations in a bout). A binomial test against the chance expectation of 50% was used to test whether the turning direction of a saccade is significantly biased in the same or opposite direction as the previous one. In further analysis of fixation durations, we divided the fixation durations in the entire corpus into those saccades just before a switch in turn direction (Pre-reversal), the fixation immediately after a switch in turn direction (Post-reversal), and all others (Within sweep). We report the number of fixations and scans in the test conditions as means with ± standard error of the mean. We also report the duration of fixations and scans in the different test conditions as means with ± standard error of the mean in milliseconds.

In comparisons across conditions, we decided to use parametric statistics despite clear departures from normality because parametric tests are said to be robust against such violations (Knief and Forstmeier 2021). We did use the Kolmogorov–Smirnov test to test if the data variables had similar distributions across the test conditions. The assumption of similar distributions was not ruled out by evidence (*p* > 0.05). Across the four test conditions in the main experiment (Left, Right, Middle, Opposite), the number of fixations per bout and scanning bouts per test and the mean durations of fixations and scanning bouts, and the intervals between scanning bouts were compared using linear and generalised linear models for the ANOVA (with mixed effects, accounting for repeated observation). The best-fit model with the lowest Akaike Information Criterion (AIC) was selected. Because the distributions in all the groups resembled Poisson distributions, we ended up using Poisson ANOVAs, ANOVAs that assume an integer “shape” parameter (Poisson-like distribution) in the groups. The between-subjects factor was the training location, Right or Left, while the repeated-measures factor was test condition, as each ant participated in all 4 test conditions. A separate generalised linear-model (Poisson) ANOVA was run to predict the training condition of the first two cohorts (Left or Right location depending on training condition) and the tests at the Far location. Training location (Left, Right, between-subjects) and test location (training location, Far, within-subjects) were the two factors. With some conditions used in two separate ANOVAs, we adopted the Holm procedure for correcting for multiple tests. The lowest p-value across the two ANOVAs was set at *p* = 0.025. If the lowest p-value of an effect fell below 0.025, the second p-value was set at 0.05. If the lowest p-value was above 0.025, the effects in both ANOVAs were considered not significant. The pairwise comparisons between the different test conditions were conducted using Tukey’s HSD test.

To understand the generation of fixations and scanning behaviours, we fitted the distributions of fixation durations and inter-scanning-bout intervals. Binning data and then fitting curves introduces errors (Sims et al. 2007). Following current practice, we used maximum-likelihood methods on the inverse cumulative frequencies of fixation-duration and inter-scanning-bout-interval distributions. These plots display in the fashion of a survival curve starting with 100% of records at 0 s and dropping to 0% at the longest duration or interval for the measure in question. For these cumulative data, we initially fitted linear and generalised linear models for the ANOVA with Gaussian and Poisson distributions, selecting the model with the lowest AIC. This test adjudicates between the Gaussian and the Poisson-like families of curves and can serve to rule out the Gaussian distribution as a model, something that looked highly unlikely simply from looking at the binned data. The inverse cumulative distributions were then fitted with three a priori plausible functions:

- Exponential (*y* = α * *e*^**βt**^), with free parameters α, β. α is a constant which scales the function. β is a free parameter estimating the negative exponent, while t represents duration or interval.
- Power law (*y* = α * t^β^) with free parameters α, β. α is a scaling constant and β is an estimate of the power exponent in the power law.
- Stretched exponential (f_β_ (t) = α * e^(–t^β^)), where ^ means “to the power of”. The approach applied in fitting a stretched exponential is similar to that used for fitting an exponential equation. The difference is that for stretched exponential problem solving, we need to pre-define a set of functions for our parameters. We set a starting value for α, β and t, which can then be adjusted to fit our data. Whereas β is the stretching parameter (0 ≤ β < 1), f_β_(t) represents the decay of observables with change in predictive parameter (differential distribution), with t representing time or duration, the predicting variable.

The best model distribution was identified objectively using the AIC.

In addition, a generalised linear mixed-model Poisson ANOVA was used to test the effect of Pre- and Post-reversal fixation durations and turn angles across all test conditions combined, with training location used as a fixed factor, and ant ID considered as a random factor. We performed Tukey post-hoc tests to evaluate whether means differed significantly between the conditions. No data were entered into multiple statistical tests in these subsidiary analyses; we thus used alpha = 0.05.

## Results

To understand scanning behaviour at the start of a homeward journey, we studied *Melophorus bagoti* foragers that had visited either the left or right feeder (Fig. 1a). Ants traversed a short channel before reaching open ground, where, before starting their way home, they exhibited much scanning.

**Fig. 3:**
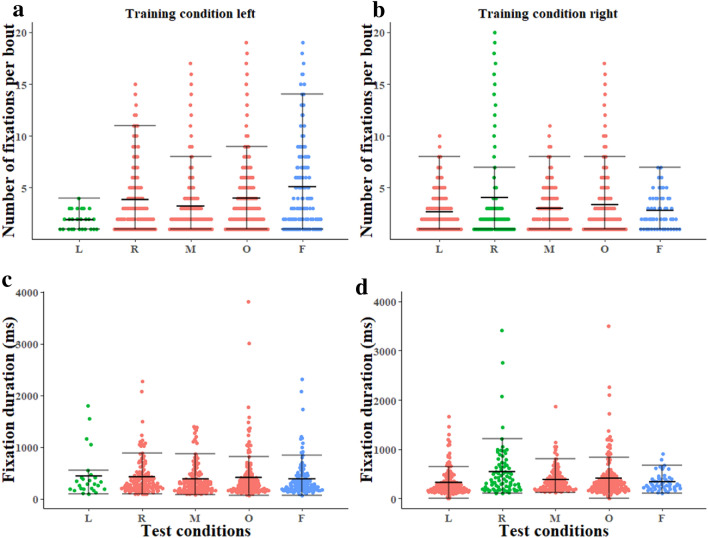
Number of fixations per bout **a**,** b** and fixation durations **c**,** d** in the Left and Right training conditions. The middle line in each test condition indicates the median, and error bars show the lower and upper quartiles in the beeswarm plots. Test locations *L*   Left, *R* Right, *M* Middle, *O*  Opposite, *F* Far. *N* 48 in Left, Right, Middle, and Opposite tests, in both Left and Right training conditions. *N* 14 for Far test, Left training; *N* 19 for Far test, Right training. In the colour scheme, green means same condition as in training, red means untrained test condition, blue means far away

We first compared scanning behaviour across test conditions. Starting with individual fixations, both the number per scanning bout and fixation durations showed pear-shaped, bottom-heavy distributions (Fig. 3), with most of the counts or durations being small in magnitude and a long skewed tail going upwards. The pattern held for all conditions. In fixation durations pooled across all conditions, about 54% fell in the range of 0–400 ms (M ± sem: 396.72 ms ± 10.67 ms). Fixation numbers were mostly similar across conditions, with the Left trained ants tested at the Left location perhaps lower (Fig. 3a, b). The generalised linear mixed-model ANOVA predicted significant differences within the test conditions (*N* = 48, *F* (3, 198) = 4.69, *p* = 0.0005, AIC: 990.2 (see Table S1A)). This effect means significant differences across individuals. The model, however, did not find significant differences across test conditions (*N* = 48, *F*(3, 198) = 0.57, *p* = 0.63) or between left and right training conditions (*F*(1, 203) = 0.05 *p* = 0.81), and the interaction of test and training conditions did not come out significant (*F*(1, 198) = 0.78, *p* = 0.37). Simultaneous pairwise comparisons using Tukey’s HSD test indicated that all the differences were statistically not significant (see Table S1B). Similarly, for the ants trained Left or Right and then tested at the Far test location (*N* = 20; Fig. 3a, b), generalised linear mixed-model ANOVA showed that training conditions (*F*(1, 22) = 0.12, *p* = 0.25), test locations (*F*(1, 20) = 0.24, *p* = 0.38), and the interaction of test and training conditions (*F*(1, 22) = 0.36, *p* = 0.57) did not have a statistically significant influence on fixation number.

In the main experiment (i.e., without the Far condition), fixation durations were also similar across test and training conditions (Fig. 3c, d). The generalized linear mixed-model ANOVA predicted significant differences within the test conditions (*N* = 48, *F* (3, 198) = 7.24, *p* = 0.0005, AIC: 2906 (see Table S2A)). The model found a non-significant test condition main effect (*N* = 48, *F*(3, 198) = 1.1, *p* = 0.35) and a non-significant training condition main effect (*F*(1, 203) = 2.03, *p* = 0.15). The interaction of test and training conditions failed to come out significant with the Holm correction (*F*(3, 198) = 4.93, *p* = 0.027). Simultaneous pairwise comparisons using Tukey’s HSD test indicated that all the differences were statistically not significant (see Table S2B). When it comes to comparing the Far tests with training-location tests on fixation durations (Fig. 3c, d), the model showed that the different test conditions (*N* = 20, *F* (1, 20) = 1.92, *p* = 0.087) did not have a statistically significant effect on fixation duration. In addition, training conditions (*F* (1, 22) = 0.66, *p* = 0.51) and the interaction of test and training conditions (*F* (1, 22) = 0.58, *p* = 0.56) both did not show significant effects.

**Fig. 4:**
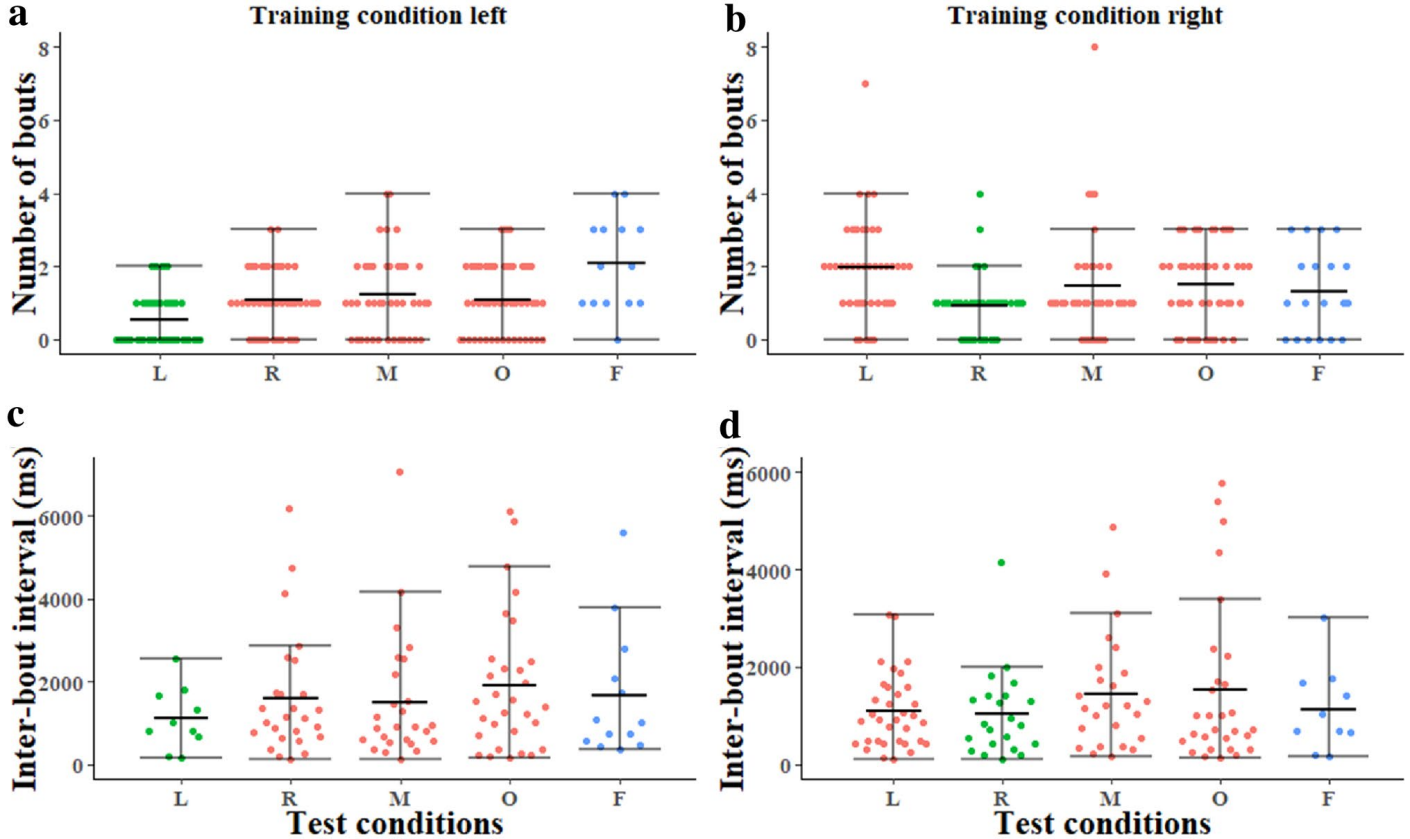
Number of scanning bouts **a**,** b** and inter-scanning-bout intervals **c**,** d** in the Left and Right training conditions. The middle line in each test condition indicates the median, and error bars show the lower and upper quartiles in the beeswarm plots. Test locations *L* Left, *R* Right, *M* Middle, *O* Opposite, *F* Far. *N* 48 in Left, Right, Middle, and Opposite tests, in both Left and Right training conditions. *N* 14 for Far test, Left training; *N* 19 for Far test, Right training. In the colour scheme, green means same condition as in training, red means untrained test condition, blue means far away

When it comes to entire scanning bouts, both the number of bouts on a trip and the inter-bout intervals also showed bottom-heavy, pear-shaped distributions (Fig. 4). The skewed tails going upwards were less prominent compared to the case of individual fixations shown in Fig. 3, especially in the temporal data for inter-event intervals. In inter-scanning-bout intervals across all conditions (M ± sem: 1672.35 ms ± 98.41 ms), 9% fell in the range of 0–800 ms and 54% fell in the range of 800–1600 ms. Some differences across conditions in the number of scanning bouts (caught on camera) were evident (Fig. 4a, b). In the main experiment, however, while the main effect within the test conditions showed significant differences (*F*(1,203) = 16.23, *p* < 0.0005), the main effect of test conditions did not show significant differences (*N* = 48, *F*(3, 198) = 0.15, *p* = 0.87, AIC = 116 (see Table S3A)). The main effect of training conditions did not show significant differences (*F*(1,203) = 0. 67, *p* = 0.41). The interaction was also not significant (*F*(3,198) = 1.17, *p* = 0.26). Simultaneous pairwise comparisons using Tukey’s HSD test indicated that all the differences were statistically not significant (see Table S3B). When it comes to comparisons with the Far tests (Fig. 4a, b), the model showed that different training conditions had a statistically significant effect on scan number (N = 20, *F*(1,22*)* = 4.8, *p* < 0.0005). Different test locations also showed significant differences in number of bouts (*F* (1,22) = 1.99, *p* = 0.04, AIC = 174.3), with ants in the Far tests displaying more scanning bouts. The interaction between training conditions and test conditions also came out significant (*F*(1,20) = 2.2, *p* = 0.02). The Left training condition had the most scanning bouts on the Far tests (Fig. 4a, blue).

The time intervals between scanning bouts seem similar across conditions and indeed did not differ significantly across test conditions or training conditions (Fig. 4c, d). The model found non-significant main effects and interaction (*N* = 48, test condition main effect: *F*(3, 99) = 0.97, *p* = 0.33; training condition main effect: *F*(1,104) = 1.88, *p* = 0.06; interaction: *F*(3,99) = 0.45, *p* = 0. 64). However, the model predicted significant differences within the test conditions (*N* = 48, *F*(3, 99) = 4.59, *p* = 0.0005, AIC: 1782.2 (see Table S4A)). Simultaneous pairwise comparisons using Tukey’s HSD test indicated that all the differences were statistically not significant (see Table S4B). A similar pattern was found when comparing Far tests with training-location tests (Fig. 4c, d). The model showed that different training conditions (*N* = 20, *F*(1,17) = 1.60, *p* = 0.21), different test conditions (*F*(1,15) = 0.36, *p* = 0.96), and their interaction (*F*(1,17) = 0.52, *p* = 0.97) all did not have a statistically significant effect on inter-scanning-bout time interval.

**Fig. 5:**
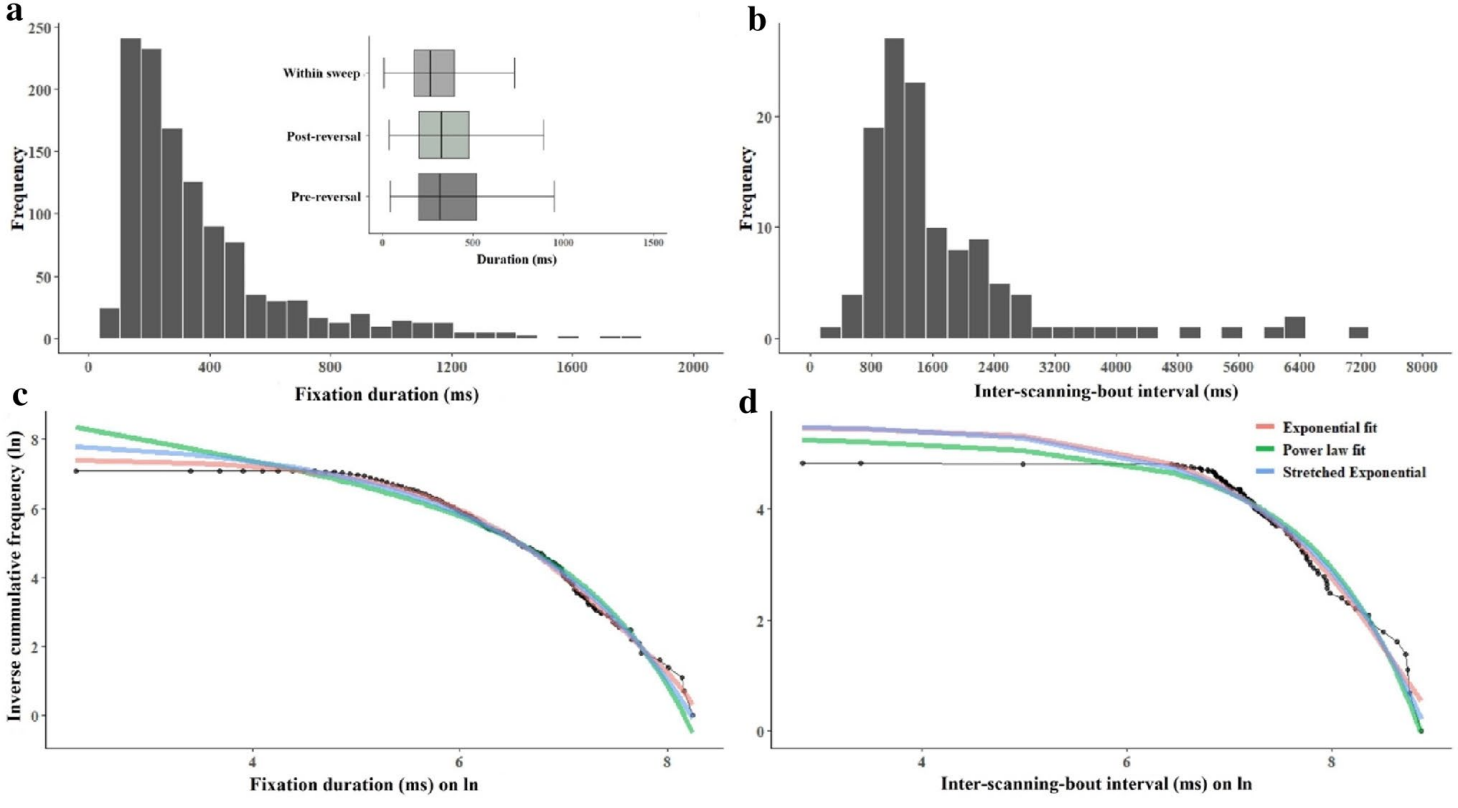
Distributions of fixation durations and inter-scanning-bout intervals. Binned frequency distributions of fixation durations **a** and inter-scanning-bout intervals **b** combining all test and training conditions. The inverse frequency distributions (in black) of fixation durations **c** and inter-scanning-bout intervals **d** combining all test and training conditions, with 3 models fitting the distributions: Power law (green), Exponential (red), Stretched Exponential (blue). The *x*- and *y*-axis measures are on a log scale. Inset in a. shows fixation durations for the fixation immediately before a change in turn direction (from turning right to turning left or from turning left to turning right, Pre-reversal), immediately after a change in turn direction (Post-reversal), or all other fixation durations (Within sweep). The boxes indicate the median and quartiles, while the whiskers show maximum and minimum. The Poisson ANOVA shows significant differences between categories in fixation durations (*F* (2, 951) = 15.17, *p* ≤ 0.0005)

We thus found no significant differences across test conditions in both the temporal parameters of inter-scanning-bout interval and fixation duration; significant differences across test conditions were found only in the numbers of such events. We then combined all conditions to have sufficient sample sizes to fit the distribution of fixation durations (Table 1, Fig. 5) and inter-scanning-bout time intervals (Table 2, Fig. 5). For both variables, a tiny proportion of very short intervals is followed by a concave decreasing gradient. The binned frequencies for these durations (Fig. 5a, b), shown for illustration only, depict shapes that are clearly not the bell shape of Gaussian distributions. The model analysis of variance favours the Poisson family of distributions over the Gaussian family for fixation intervals (*F* = (1, 141) = 22.56, *p* = 0.0005; Akaike Information Criterion (AIC) = 148, AIC Weight = 0.99 for the Poisson and AIC = infinite, and AIC Weight = 0.01 for the Gaussian), inter-scanning-bout intervals (*F* = (1, 103) = 12.13, *p* = 0.0005; AIC = − 12.8, AIC Weight = 0.95 for the Poisson and AIC = 496, AIC Weight = 0.05 for the Gaussian. A similar result favouring the Poisson family was found for scanning-bout durations (shown in Fig. S1), with the Akaike weights leaning heavily towards the Poisson family (*F* = (1, 275) = 12.4, *p* = 0.0005; AIC = − 67.3, AIC Weight = 1 for the Poisson and AIC = 243, AIC Weight = 0 for the Gaussian). Curve fits based on maximum likelihood were then conducted on inverse cumulative distributions, using the power law, exponential, and stretched exponential distributions (Figs. 5c, d, S2). For all three temporal measures, fixation durations, inter-scanning-bout intervals, scanning-bout durations, the AICs favour the exponential function overwhelmingly as providing the best fit; the exponential reaped most of the AIC weights (Tables 1, 2, and S5).

**Table 1:** The performance of curve fits of fixation durations across all testing and training conditions combined

Curve fit	AIC	AIC weight	*R* squared	*P*-value
Power law	− 35.47824	0.0	0.9720799	2.2e-16
Exponential	− 552.5975	1	0.9953388	2.2e-16
Stretched exponential	− 313.4917	0.0	0.972809	5.453e-06

*AIC* Akaike Information Criterion (the more negative, the better the model). Curve fits were performed using the maximum-likelihood method on the inverse cumulative distribution (Fig. 5c)

**Table 2:** The performance of curve fits of inter-scanning-bout intervals across all testing and training conditions combined

Curve fit	AIC	AIC weight	*R* squared	*P*-value
Power law	− 12.15732	0.0	0.9503521	< 2.2e-16
Exponential	− 67.40918	0.99	0.9712195	< 2.2e-16
Stretched exponential	− 36.99868	0.0	0.961	5.551e-06

*AIC* Akaike Information Criterion (the more negative, the better the model). Curve fits were performed using the maximum-likelihood method on the inverse cumulative distribution (Fig. 5d)

### Subsidiary analyses

Ants sometimes turned only in one direction within a scanning bout and sometimes they switched direction (turn right after turning left or turn left after turning right) within a bout. Fixations were divided according to whether they occurred just before a switch in turn direction (Pre-reversal: 474.8 ms ± 28.4 ms in duration), immediately after a switch in turn direction (Post-reversal: 440.4 ms ± 17.2 ms), and all others (Within sweep: 353.6 ms ± 14.07 ms). The durations of these different categories of fixations differed significantly (*F* (2, 951) = 15.17, *p* ≤ 0.0005, Fig. 5a inset; Fig. S3), with the Pre- and Post-reversal fixation durations being longer. The post hoc comparisons of means across conditions found significant differences between the Within sweep and Pre-reversal durations (*F(*1, 946) = 3.63, *p* = 0.026) and between the Pre-reversal and Post-reversal durations (*F(*1, 946) = 0.78, *p* < 0.0005), but no significant difference between the Within sweep and Post-reversal durations (*F(*1, 946) = 15.4, *p* = 0.06). The analysis also found a significant individual factor (*F* (34, 245) = 12.66, *p* < 0.0005), meaning that individual ants varied in this parameter.

As a second subsidiary analysis of the distribution of behaviours, we examined the duration of entire scanning bouts, that is, from the time that an ant stopped to take a fixation to the time that it resumed walking (Fig. S1, Table S5), a measure that must correlate with the number of fixations within a bout. Initially, we checked whether the scanning-bout duration differed across the training and test conditions. The generalized linear mixed-model ANOVA predicted significant differences within the test conditions in scanning-bout durations (*N* = 48, *F* (3, 313) = 4.65, *p* = 0.0005, AIC = 5457.6 (see Table S6A)). The model, however, did not find significant differences across test conditions (*N* = 48, *F*(3, 313) = 0.93, *p* = 0.47) or the Left and Right training conditions (*F*(1, 315) = 0.09, *p* = 0.34), and the interaction of test and training conditions also did not come out significant (*F*(3, 313) = 0.4, *p* = 0.68). Simultaneous pairwise comparisons between the different test conditions conducted using Tukey’s HSD test indicated that all the differences were statistically not significant (see Table S6B). Similarly, for the ants trained Left or Right and then tested at the Far test location (*N* = 20), generalised linear mixed-model ANOVA showed that training conditions (*F*(1, 41) = 0.44, *p* = 0.65), test locations (*F*(1, 39) = 1.1, *p* = 0.38), and the interaction of test and training conditions (*F*(1, 39) = 0.21, *p* = 0.83) all did not have a statistically significant influence on scanning-bout duration.

**Fig. 6:**
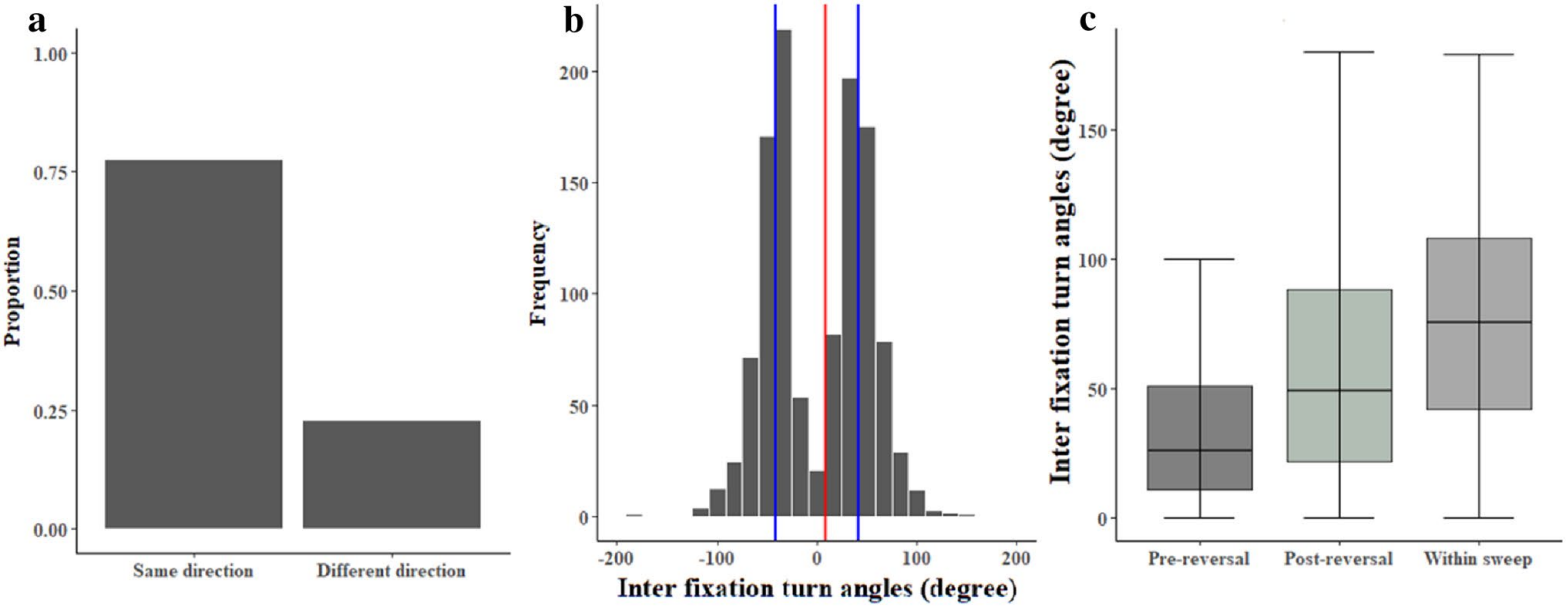
The turning direction and turn angles of fixations. **a** The proportion of fixations turning to the same direction or different direction from the previous fixation. **b** The frequency of the inter-fixation turn angles at different angular ranges across all test and training conditions. The red line shows the median turn angle for the entire distribution, while the blue lines show medians for the negative (all values < 0) and positive (all values > 0) halves of the distribution. **c** Turn angles of the turn immediately before a reversal of turn direction (Pre-reversal), immediately after a change in turn direction (Post-reversal), and all other turns (Within sweep). The boxes indicate the median and quartiles, while the whiskers show maximum and minimum

In another secondary analysis, we examined the properties of turns within a scanning bout. Successive saccades tend to turn in the same direction (Fig. 6a). A binomial test against the chance expectation of 50% showed that fixation turning directions are significantly biased to be in the same direction as the previous one (*p* < 0.005). For bouts with directional switches in turn direction of fixations, we further examined when the ants switched directions, measured in terms of the proportion of time into the entire duration of a bout (Fig. S4). Across an otherwise Gaussian-looking distribution, two peaks stand out: one at the middle of the bout and one right at the end.

Regarding the magnitude of turns, 57% of inter-fixation turn angles fell in the range of ± 45º (Fig. 6b). The distribution is clearly bimodal, with similar sub-distributions to the right and left of 0. The overall median of all turns came out close to 0 (8.5°, measured to the nearest half a degree), while the right half and the left half of the distribution (dividing at 0°) were both close to a quarter turn, + 42° median on the right and –41° on the left. These averages hide some systematic differences in turn angles when the ant changed turn directions from right to left or from left to right. We again separated turns into those just before a directional switch, just after a directional switch, or within a sweep in the same direction (the rest). The ants made on average biggest turns within a sweep of turns in the same direction and smallest turns just before a switch in turn direction (Fig. 6c). The ANOVA shows significant differences between categories in turn angles (*F*(2, 949*)* = 4.58, *p* < 0.0005). The post hoc comparisons of means revealed a significant difference between the Within sweep and Pre-reversal conditions (*F(*1, 949) = 10.94, *p* ≤ 0.008) in turn angles, whereas the Within sweep vs. Post-reversal turn angles (*F(*1, 949) = 0.94, *p* = 0.06) and the Pre-reversal vs. Post-reversal turn angles (*F(*1, 949) = 0.78, *p* = 0.34) did not show any significant differences.

## Discussion

We examined the distribution in time of the scanning behaviours that desert ants produce before they set off on their journey home from various locations. In particular, we examined the distribution of fixation durations and inter-event intervals between the start of successive scanning bouts, each bout consisting of one or, typically, more fixations in different directions. The cumulative distributions of both these temporal parameters are exponential, with the exponential function providing by far the best fit. An exponential distribution is a signature of a Poisson or random-rate process: at every moment in time, there is a constant probability of an event, either stopping the fixation and going on to the next behaviour (such as another saccade or the resumption of walking) or, in the case of scanning bouts, the start of such a bout. No other plausible process generates such distributions in durations or intervals. This means that some process, presumably in the brain of the ant, is generating random-rate outputs that control these scanning behaviours. In further subsidiary analyses, and in contrast to this randomness, we found regularities in the turning angles of body saccades in a scanning bout: both a left turn and a right turn showed notable peaks around 45° and some regularities are found in the directions of turns (right or left) as well. We focus our discussion on these two major points but begin with some background on the motifs of ant paths on which scanning bouts are superimposed.

### The neural basis for control of ant path motifs

The key insect brain regions that are involved in navigation are the mushroom bodies (MB) and the central complex (CX) (Webb and Wystrach 2016; Heinze 2017). The MB play a role in the appetitive and aversive associative learning of stimulus–reward contingencies and have recently been shown to be essential for view-based navigation in two species of ants (Buehlmann et al. 2020; Kamhi et al. 2020). Current working models of MB function posit that after the learning of view-based information for navigation, they are able to produce an output that relates to the familiarity of an experienced view (Baddeley et al. 2012; Ardin et al. 2016). This information can be assigned a valence or associated with turn information (Le Möel and Wystrach 2020; Wystrach et al. 2020b) and would be a signal available to other brain regions that might be used to modulate motor patterns based on visual familiarity or reliability of visual information.

The CX has been implicated in the control of speed and turning and the maintenance of heading information (review: Honkanen et al. 2019) and the output regions of the CX are the lateral accessory lobes (LAL) (reviews: Namiki and Kanzaki 2016; Steinbeck et al. 2020), which are conserved brain areas implicated in sensorimotor control across a range of insect species. The LAL have a bilateral organisation that separately collates sensory information from the left and right hemisphere of the insect brain. Ipsilateral descending neurons are capable of passing on information directly to non-brain motor centres, such as for sensory-driven steering. But the LAL also contain contra-lateral neurons that might be capable of forming a central pattern-generating (CPG) circuit across the two hemispheres of the LAL. This circuit could be used to generate phasic motor outputs underpinning oscillatory movements, such as those associated with the search for sensory signals in some insects (Namiki and Kanzaki 2016; Steinbeck et al. 2020). Thus, the LAL represent the likely neural substrate for the generation of oscillatory patterns during ordinary ‘wiggling’ ant paths as well as scanning bouts.

The output of these oscillators in ants is regular lateral turns to the left and right (Murray et al. 2020; Clement et al. 2023). Other oscillatory patterns of movements are found in other organisms (Namiki and Kanzaki 2016; Wystrach et al. 2016; Cheng 2022). These rhythms are internally, endogenously generated, but are still subject to modification by external circumstances. That is, servomechanisms could adjust parameters of oscillations such as their frequency or amplitude (Cheng 2022). Thus, aversive encounters could serve to increase the extent of oscillations (called “meander”) the next time an ant nears the place of an aversive encounter (Wystrach et al. 2019, 2020a). The modulation of features of the basic rhythm is thought to provide balance between exploration for useful sensory information and exploitation of that sensory information to navigate towards a goal (Clement et al. 2023). The LAL might orchestrate both the exploitation of sensory information and the exploration for reliable sensory cues. Agent-based simulations have given existence proof that view-based navigation can be implemented successfully where the amplitudes of path oscillations are modulated by a visual familiarity signal (Kodzhabashev and Mangan 2015; Le Möel and Wystrach 2020), as observed in ants (Murray et al. 2020; Clement et al. 2023).

### Explaining random-rate processes in scanning bouts and fixations

We have shown here that the temporal distribution of scanning bouts is best described as being produced by a random-rate process, which suggests a single parameter or signal is responsible for the rate of their production. Scanning bouts, however, are less likely to occur in familiar surrounding (Fig. 4, green), suggesting that this parameter can be modulated. Given what we currently know about the neural basis of navigation, the best explanation would be that a visual familiarity signal from the MB was able to switch the LAL circuit between an ordinary, visually guided ‘wiggly’ sinusoidal path (e.g., Clement et al. 2023) and, when visual familiarity is lower than a threshold, a scanning bout. We suppose that at the beginning of a trip, the visual (un)familiarity threshold is often reached, with inherent noise in the neural familiarity signal pushing the familiarity measure occasionally below threshold. In line with the data, we further suppose that this noise reaches threshold as a random-rate process, thus producing the exponential distribution of inter-scanning-bout intervals. Neural processes, in single neurons at least, often operate with Poisson spiking processes (Sanger 2003; Lawlor et al. 2018).

The exponential distribution of fixation durations means that the ending of fixations is also produced by some random-rate process. We envisage a similar threshold-crossing process that governs when a fixation ends. We suppose that the random-rate process governs when enough information has been gathered to reach a neural threshold indicating sufficient familiarity gained from a fixation. As we found systematic variations across sweeps of fixations, discussed further below, this thresholding process, however it is realized neurobiologically, is subject to modulation. We envisage modulation adjusting the rate parameter, perhaps by adjusting threshold level. Our discussion here is highly speculative and points to the need for much more neurobiological investigation targeting this issue.

A second tentative possibility requiring much future confirmatory evidence is that local thoracic networks may trigger occasional stops (the beginning of a scanning bouts), and the probability of occurrence could be modulated by descending neurons conveying information about current certainty (such as visual familiarity, or changes in odour concentration during chemotaxis). Meanwhile, the intrinsically generated oscillations triggering left and right turns are always running, as seems to be the case here with ants. This hypothesis has some support in *Drosophila* larvae (Berni et al. 2012; Wystrach et al. 2016; Loveless et al. 2019).

### Explaining regularities in scans

Within a scanning bout, the control of saccade direction and amplitude appears to be under the control of a slower, intrinsic oscillatory process as described by Clement et al. (2023). First, the direction of a given saccade is more likely to be in the same direction as the previous one, suggesting the existence of a longer-lasting process. Second, the duration of a full sequence of saccades in the same direction fits with the duration of a full sweep on one side of an oscillatory cycle as observed in several species of ants (around 1 s on average, that is, 0.5 Hz for a full oscillatory cycle (Clement et al. 2023; Wystrach et al. 2016; Fig. S1). Third, variation in this duration is significantly explained by ants’ individuality in fixation durations, showing the existence of a conserved rhythm within individuals that is conserved across trials and conditions. Fourth, saccades’ turn angles are bigger and pauses between saccades are shorter in the middle of a sweep, compared with saccades close to a reversal of direction. This fits with what is observed within the intrinsic oscillatory cycle: angular speed rises to a peak in the middle of a sweep and slows down to zero (with often a real pause) during reversal (Clement et al. 2023). It thus seems that the drive for turning left and right—both when walking and within scanning bouts—is under the control of the same intrinsic oscillatory rhythm, but that initiation and ending of a scanning bout are triggered by a parallel, random-rate process, independently of the current oscillatory phase.

Ultimately, these questions concerning the initiation of scanning bouts and the characteristics of such bouts are likely to be answered in full only when we are able to monitor neural activity in key circuits of behaving individuals. Two of the biggest gaps in our account are: (1) what the nature of a familiarity signal is, cognitively and neurobiologically, and (2) how information is transferred from the mushroom bodies to the central complex (see Wystrach et al. 2020b for evidence of such a transfer of information).

It remains to be explained why scanning bouts are composed of sequential fixations rather than a smooth sweep left and/or right as observed in fly larvae. Functionally, regular fixations during scanning bouts do guarantee a stabilized view, which must increase the accuracy of visual recognition by preventing motion blur. Body saccades show some stereotypicality in the amount of turn on each saccade. Although a finer analysis is needed, these body saccades can perhaps be described as modal action patterns. The anatomy and physiology of the ant’s body likely limits how far an ant can rotate in one action within a body saccade. The distributions of turn angles, however, suggest that average turns, with peaks just under 45° (Fig. 6b), are not near that maximum, with tails well exceeding 45°. Regarding the near − 45° average, we note that 8 directionally tuned units have been posited to undergird the compass signal in insects (Pfeiffer and Homberg 2014; Stone et al. 2017). Whether these two pieces of data are related as well as the kinematics of turns could certainly use more empirical and theoretical analysis.

## Conclusion

We examined the timing of the initiation of scanning bouts at the beginning of the red honey ant’s (*M. bagoti*) trip home and the distribution of the durations of individual fixations. We found exponential distributions in inter-event times in both cases, implying underlying Poisson or random-rate processes generating these behaviours. We suppose that different forms of intermittent reaching of thresholds trigger these behaviours, the intermittent nature stemming from random fluctuations in underlying neural processes representing visual familiarity, however that is coded in an ant’s brain. Much more evidence is needed to support our tentative interpretations, especially neurobiological evidence, but this study shows that the neuroethological community should take on board the study of the timing of behaviours (Cheng 2022).

## Supplementary Information

Below is the link to the electronic supplementary material.

Supplementary file1 (DOCX 153 KB)

## Data Availability

Analysis scripts and data files are available at Open Science framework: https://osf.io/e7qag/.
