# Supplementary material for: Scanning behaviour in ants: an interplay between random-rate processes and oscillators
Source: J Comp Physiol A Neuroethol Sens Neural Behav Physiol. 2023 Apr 24;209(4):625–39. doi: 10.1007/s00359-023-01628-8 (PMC10354138; doi:10.1007/s00359-023-01628-8)
Supplement: Supplementary file 1 — Supplementary file1 (DOCX 153 KB) [file 359_2023_1628_MOESM1_ESM.docx]

Scanning behaviour in ants: an interplay between random-rate processes and oscillators

Sudhakar Deeti^1^, Ken Cheng ^1^, Paul Graham^2^, Antoine Wystrach^3^

^1^School of Natural Sciences, Macquarie University, Sydney

^2^ School of Life Sciences, University of Sussex, Brighton

^3^ Centre de Recherches sur la Cognition Animale, CBI, CNRS, Université Paul Sabatier, Toulouse

**Supplementary figures and tables**


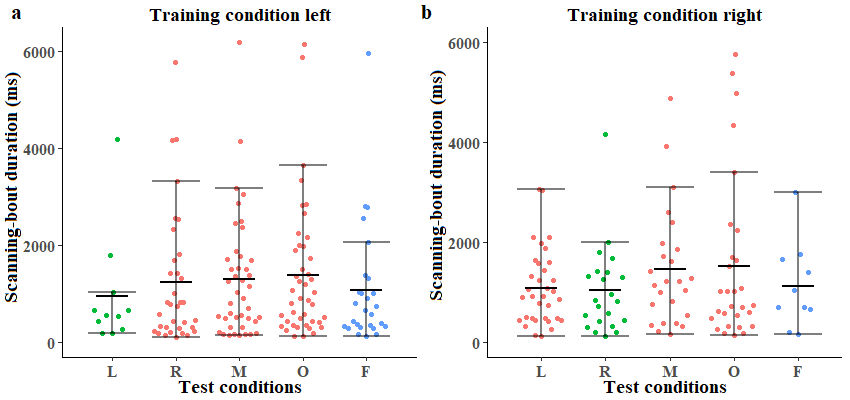


**Fig. S1** Scanning-bout durations (**a**, **b**) in the Left and Right training conditions. The middle line in each test condition indicates the median, and error bars show the lower and upper quartiles in the beeswarm plots. Test locations: L = Left, R = Right, M = Middle, O = Opposite, F = Far. N = 48 in Left, Right, Middle, and Opposite tests, in both Left and Right training conditions. N = 14 for Far test, Left training; N = 19 for Far test, Right training. In the colour scheme, green means same condition as in training, red means untrained test condition, blue means far way


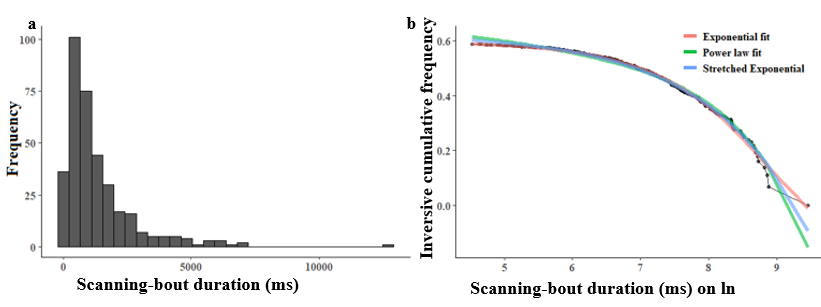


**Fig. S2** Distribution of durations of entire scanning bouts. Binned frequency distributions of scanning-bout durations (**a**) combining all test and training conditions. The inverse frequency distributions (in black) of scanning-bout durations (**b**) combining all test and training conditions, with 3 models fitting the distributions: Power law (green), Exponential (red), Stretched Exponential (blue). The *x*- and *y*-axis measures are on a log scale


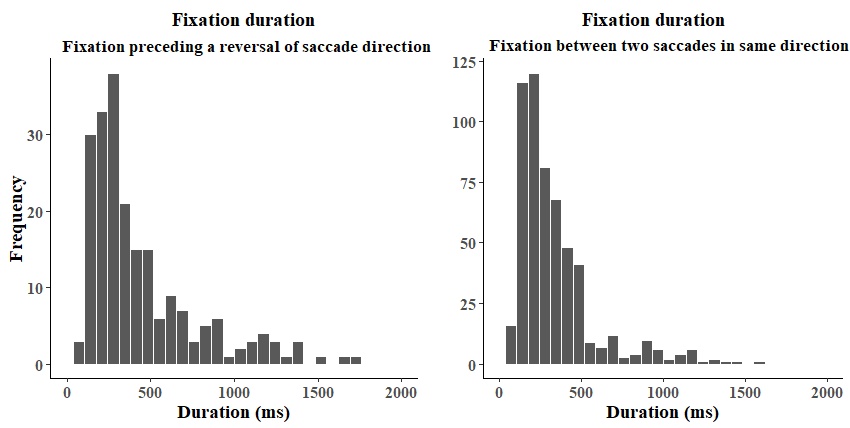


**Fig S3** The duration of fixations in different categories of fixations within a scanning bout. The binned frequencies of the duration of fixations preceding a reversal in the direction in which a saccade turns (Left) and fixation durations of saccades turning in the same direction as the previous saccade (Right)


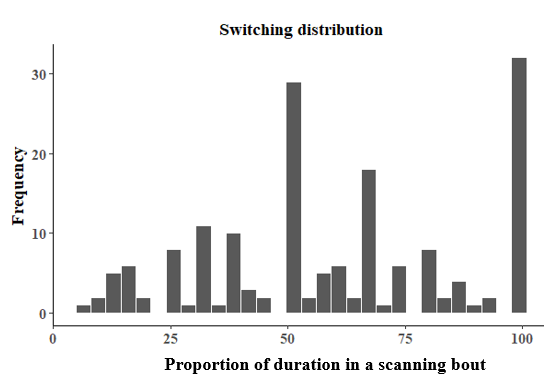


**Fig. S4** Pooling all test conditions, frequencies of the times within a scanning bout at which a switch in turning direction of a body saccade took place, measured in terms of proportion of time into each scanning bout

**Table S1A and S1B** (**A**) Linear and Generalized linear model AIC and AIC weight report and (**B**) Post-hoc test report for number of fixations, comparing pairs of test conditions

**A**

| Model | AIC | R_squared | AIC weight |
| --- | --- | --- | --- |
| LM | Inf | 0.01052865 | 0 |
| GLM | 990.202 | 0.01081076 | 1 |

**B**

|  | diff | lwr | upr | p adj |
| --- | --- | --- | --- | --- |
| M–L | 0.4358501 | -0.9919805 | 1.8636807 | 0.8585557 |
| O–L | 0.6121573 | -0.7651823 | 1.9894969 | 0.6580860 |
| R–L | 0.2060915 | -1.2351721 | 1.6473551 | 0.9825976 |
| O–M | 0.1763072 | -1.1453752 | 1.4979896 | 0.9857768 |
| R–M | -0.2297586 | -1.6179303 | 1.1584132 | 0.9734819 |
| R–O | -0.4060658 | -1.7422486 | 0.9301171 | 0.8601503 |

Note. L = Left test, M = Middle test, R = Right test, O = Opposite test.

**Table S2A and S2B** (**A**) Linear and Generalized linear model AIC and AIC weight report and (**B**) Post-hoc test report for fixation durations, comparing pairs of test conditions

**A**

| Model | AIC | R_squared | AIC weight |
| --- | --- | --- | --- |
| LM | Inf | 0.03302362 | 0 |
| GLM | 2906.4 | 0.03577737 | 1 |

**B**

|  | Diff | lwr | upr | p adj |
| --- | --- | --- | --- | --- |
| M–L | 40.79919 | -117.15124 | 198.7496 | 0.9085885 |
| O–L | 58.03227 | -94.33270 | 210.3972 | 0.7571581 |
| R–L | 117.04819 | -42.38822 | 276.4846 | 0.2304977 |
| O–M | 17.23309 | -128.97493 | 163.4411 | 0.9900909 |
| R–M | 76.24901 | -77.31424 | 229.8123 | 0.5726617 |
| R–O | 59.01592 | -88.79619 | 206.8280 | 0.7294676 |

Note. L = Left test, M = Middle test, R = Right test, O = Opposite test.

**Table S3A and S3B** (**A**) Linear and Generalized linear model AIC and AIC weight report and (**B**) Post-hoc test report for number of scanning bouts, comparing pairs of test conditions

**A**

| Model | AIC | R_squared | AIC weight |
| --- | --- | --- | --- |
| LM | 1245.6 | 0.1490533 | 0 |
| GLM | 1169 | 0.09028545 | 1 |

**B**

|  | diff | lwr | upr | p adj |
| --- | --- | --- | --- | --- |
| M–L | -0.4255793 | -2.642017 | 1.7908587 | 0.9595689 |
| O–L | 0.2803030 | -1.857757 | 2.4183632 | 0.9864774 |
| R–L | -1.2435065 | -3.480797 | 0.9937836 | 0.4759947 |
| O–M | 0.7058824 | -1.345780 | 2.7575452 | 0.8093530 |
| R–M | -0.8179272 | -2.972802 | 1.3369478 | 0.7590879 |
| R–O | -1.5238095 | -3.597982 | 0.5503626 | 0.2299250 |

Note. L = Left test, M = Middle test, R = Right test, O = Opposite test.

**Table S4A and S4B** (**A**) Linear and Generalized linear model AIC and AIC weight report and **(B**) Post-hoc test report for inter-scanning-bout intervals, comparing pairs of test conditions

**A**

| Model | AIC | R_squared | AIC weight |
| --- | --- | --- | --- |
| LM | 58694 | 0.09028545 | 0 |
| GLM | 1782.2 | 0.08130338 | 1 |

**B**

|  | diff | lwr | upr | p adj |
| --- | --- | --- | --- | --- |
| M–L | -39.06989 | -849.0686 | 770.9288 | 0.9992805 |
| O–L | 512.66434 | -212.7100 | 1238.0387 | 0.2580229 |
| R–L | 77.40447 | -906.9767 | 1061.7856 | 0.9969144 |
| O–M | 551.73423 | -229.0803 | 1332.5488 | 0.2581929 |
| R–M | 116.47436 | -909.4445 | 1142.3932 | 0.9908746 |
| R–O | -435.25988 | -1395.7700 | 525.2502 | 0.6384522 |

Note. L = Left test, M = Middle test, R = Right test, O = Opposite test.

**Table S5** The performance of curve fits of scanning-bout durations across all testing and training conditions combined. AIC = Akaike Information Criterion (the more negative, the better the model). Curve fits were performed using the maximum-likelihood method on the inverse cumulative distribution (Fig. S2b)

| **Curve fit** | **AIC** | **R_Squared** | **AIC Weight** | **P_Value** |
| --- | --- | --- | --- | --- |
| Power Law | -308.788 | 0.983 | 0 | < 2.2e-16 |
| Exponential | -664.8378 | 0.995 | 1 | < 2.2e-16 |
| Stretched Exponential | -509.9836 | 0.992 | 0 | <2e-16 |

**Table S6A and S6B** (**A**). Linear and Generalized linear model AIC and AIC weight report and (**B**) Post-hoc test report for scanning-bout durations, comparing pairs of test conditions

**A**

| Model | AIC | R_squared | AIC weight |
| --- | --- | --- | --- |
| LM | Inf | 0.008864817 | 0 |
| GLM | 5457.614 | 0.008864817 | 1 |

**B**

|  | Diff | lwr | upr | p adj |
| --- | --- | --- | --- | --- |
| M–L | -164.24242 | -772.6759 | 444.1911 | 0.8980842 |
| O–L | 52.14576 | 524.0723 | 628.3638 | 0.9954953 |
| R–L | -131.68831 | -773.0336 | 509.6569 | 0.9516502 |
| O–M | 216.38818 | -359.8299 | 792.6063 | 0.7666208 |
| R–M | 32.55411 | -608.7911 | 673.8994 | 0.9991938 |
| R–O | -183.83407 | -794.7020 | 427.0339 | 0.8646881 |

Note. L = Left test, M = Middle test, R = Right test, O = Opposite test.
